# Long‐Term Effect of Acetylcholinesterase Inhibitors on Behavioral and Psychological Symptoms of Dementia

**DOI:** 10.1002/gps.70195

**Published:** 2026-01-22

**Authors:** Giovanni Zuliani, Francesca Boscolo Bragadin, Tommaso Romagnoli, Michele Polastri, Carlo Cervellati, Francesco di Paola Dario, Gloria Brombo, Marco Zuin

**Affiliations:** ^1^ Department of Translational Medicine and for Romagna University of Ferrara Ferrara Italy

**Keywords:** acetylcholinesterase inhibitors, alzheimer's disease, behavioral and psychological symptoms of dementia, cognitive decline, dementia

## Abstract

**Objective:**

Behavioral and psychological symptoms of dementia (BPSD) are critical aspects of the clinical presentation of dementia. There is no universally accepted approach for the managment of BPSD, currently based first on a non‐pharmacological and subsequently on a pharmacological approach. We explored the potential effect of long‐term treatment with acetylcholinesterase inhibitors (AChEI) on BPSD severity over time.

**Methods:**

The initial sample included 4032 older patients with mild‐moderate dementia (Alzhemier's disease ‐ AD, Lewy body dementia ‐ LBD, or vascular dementias ‐ VaD) from the National Alzheimer's Coordinating Center Uniform Data Set (NACC UDS). After propensity score matching, a cohort of 1408 patients (704 treated with AChEI = AChEI+ and 704 not treated = AChEI−) was generated. The mean age was 73.2 years (females: 50.4%). The mean follow‐up duration was 4.3 ± 1.6 years (range: 2.2–8.3 years). Patients were evaluated at baseline, T1 (2 years), T2 (4 years), T3 (6.2 years), and T4 (8.1 years). BPSD severity was assessed by Neuropsychiatric Inventory (NPI‐Q).

**Results:**

The baseline mean NPI‐Q severity score was 1.33. At T4, the score increased to 1.41 in AChEI− patients (+6% from baseline), while it decreased to 1.26 in AChEI+ (−6%) (all *p* < 0.01 from T1 to T4). As regards the NPI‐Q sub‐items, six of them (hallucinations, agitation/aggression, depression/dysphoria, anxiety, disinhibition and irritability/lability) exhibited significant differences over time (all *p* < 0.01) in favor of the AChEI + group (stabilization or improvement). Similar trends were observed when LOAD, LBD and VaD were considered separately. In contrast, for five domains (delusions, elation/euphoria, motor disturbances, night‐time behaviors, and appetite/eating changes) no differences were observed.

**Conclusions:**

Our study supports the potential role for AChEI in BPSD management, demonstrating a trend toward symptoms stabilization or improvement in patients with mild‐moderate dementia. Although the effects were not uniform across all NPI‐Q domains, and the limitations of the study, our results reinforces the relevance of AChEI in the comprehensive treatment of dementia.

## Key points

- Acetylcholinesterase inhibitors (AChEI) are the first line drugs currently used for the treatment of patients with mild/moderate dementia due to Alzheimer's disease (AD)
- Behavioral and psychological symptoms of dementia (BPSD) are critical components of the clinical presentation of dementia
- AChEI appeared to exert beneficial effects on some BPSD, particularly on agitation, depression, anxiety, irritability, and hallucinations
- AchEI, in addition to acting on cognitive symptoms, appear to alleviate BPSD severity

## 1 Introduction

Behavioral and psychological symptoms of dementia (BPSD) are critical components of the clinical presentation of dementia. Per se, BPSD are strongly associated with reduced quality of life for both patients and caregivers, increased levels of disability, greater rate of institutionalization, prescription of psycothropic medications, and higher overall economic costs related to the disease. In institutional settings, BPSD also heightens the risk of physical or pharmacological restraint, and can negatively affect the well‐being of other patients. Managing BPSD is often challenging due to their complex pathophysiology and the high prevalence of multiple comorbidites in patients with dementia. The neurobiological mechanisms underlying BPSD remain poorly understood, and research on specific biomarkers is limited, hindering the development of targeted therapies. To date, there is no universally accepted approach for the managment of BPSD [1, 2]. Non‐pharmacological interventions are generally reccomended as the first‐line strategy. These include caregiver training, addressing underlying medical conditions, implementing behavioral and enviromental modifications, etc. [3]. Certain non‐pharmachological interventions, such as music therapy, sensory stimulation, and dementia care mapping have demonstratated effectiveness in managing agitation and related symptoms [4]. However, in situations where BPSD pose a risk to patients or others, or cause significant distress to patients or caregivers, pharmachological treatment may be necessary. Unfortunately, the use of antiphsychotics for BPSD has been linked to increased morbidity and higher overall mortality rates [5, 6], underscoring the need for alternative therapies with a more favorable profile.

Currently, acetylcholinesterase inhibitors (AChEI) are the standard treatment for cognitive symptoms in Alzheimer's disease (AD) [7], and are also used in other forms of dementia, including vascular dementia (VaD) and Lewy body disease (LBD) [8, 9]. Previous studies have reported a potential association between AChEI use and improvements in BPSD, as measured by the Neuropsychiatric Inventory Questionaire (NPI‐Q) [10]. However, it remains unclear which specific NPI domains may be most affected and whether improvements translate into meaningful clinical benefits [10].

This study aims to explore the potential association between long‐term AChEI therapy and changes in the severity of BPSD over time, by analyzing data from patients with mild to moderate dementia enrolled into the National Alzheimer's Coordinating Center Uniform Data Set (NACC UDS).

## 2 Materials and Methods

Data have been obtained from the NACC UDS, which is a nationwide repository for longitudinal data collected from approximately 34 current or previously NIA funded Alzheimers disease Research Centers (naccdata.org). Each patient entered the NACC registry at the time of the diagnosis of dementia and possible initiation of treatment. Local ethics committees at each center approved the study, and all participants provided written informed consent. All procedures were conducted in accordance with applicable guidelines and regulations.

For the purposes of this study, participants were eligible for inclusion if they were 65 years of age or older at the time of their initial visit, and had received a clinical diagnosis of dementia. Accepted dementia subtypes included late‐onset AD (LOAD), VaD, and LBD. To ensure adequate longitudinal data, participants were required to complete an initial evaluation and at least two subsequent in‐person follow‐up visits within a 2‐year period, each of which included a Mini‐Mental State Examination (MMSE). Additionally, only individuals with a baseline MMSE score of 10 or higher were considered for inclusion. Participants were excluded from the study if they had fewer than three consecutive in‐person visits with MMSE assessments, were receiving memantine therapy, or presented with a baseline MMSE score below 10, reflecting severe dementia.

### 2.1 Dementia Definitions

Dementia was defined as meeting criteria for AD, VaD or LBD [11, 12, 13] defined as: (1) objective cognitive impairment (i.e., performances falling greater than 1.5 standard deviations outside the age‐adjusted normative mean) in at least two cognitive systems (memory, language, attention or executive functioning), and (2) cognitive impairment contributes directly to impaired activities of daily living.

### 2.2 NPI Evaluation

The NPI‐Q was designed to assess BPSD associated with AD and other forms of dementia. This instrument is frequently used in experimental studies, where neuropsychiatric data collection is of fundamental importance [14]. The NPI‐Q includes 10 behavioral and two neurovegetative areas: Delusions, Hallucinations, Agitation/Aggression, Depression, Anxiety, Euphoria, Apathy, Disinhibition, Irritability, Aberrant Motor Behavior, Night‐time Behavior Disturbances, Appetite and Eating Disturbances [14]. The NPI‐Q is based on the observations of an informed caregiver. The questions focus on the patient's behavioral changes that have occurred since the onset of the disease (lifelong behaviors are not considered). Frequency (how often the most problematic behaviors occur) and severity (how disturbing or disabling the behaviors analyzed are) are assessed. In the present study (based on NACC UDS database) the NPI‐Q was used, which evaluates only the severity of BPSD. This was classified as follows: 0: no disorder; 1: disorder present with mild change; 2: disorder present with non‐serious change; 3: disorder present with severe/dramatic change. The NPI‐Q and its sub‐items were therefore expressed as severity of presentation by means of a score ranging from 0 (absent) to 3 (maximum severity).

### 2.3 Statistical Analysis

Pearson Chi‐square and Wilcoxon rank‐sum tests for the pre‐matched population, and McNemar's test and paired sample *t*‐test for the matched population were used to compare baseline characteristics between the two groups as appropriate.

Based on the propensity score matching (PSM) method, the clinical baseline data of the two groups were balanced, and the regression model variables included age, sex, previous diabetes mellitus (DM), hypertension, stroke, smoking, alcohol consumption, systolic blood pressure (SBP), diastolic blood pressure (DBP) and heart rate of the patients after inclusion. The propensity score of each patient was calculated by the 1:1 nearest matching method, and caliper matching was employed to limit the logarithmic standard deviation of the propensity score to 0.10 to prevent the difference between each pair of matched individuals. Based on this, a propensity score‐matched cohort of 1.408 patients (704 AChEIs + and 704 AChEIs−) was generated, using a nonparsimonious multivariable logistic regression model to calculate, for each patient, a propensity score, using as covariates: age at baseline (years), sex, years of formal education, dependence level, requirement of assistance for complex and basic activities, dementia type, comorbidities (atrial fibrillation, stroke/TIA, hypertension, diabetes, hypercholesterolemia, thyroid disease, depression in the last two years, urinary incontinence), therapies (anti‐adrenergic agents, anxiolytic agents, anti‐hypertensive drugs, antidepressants, antipsychotic agents, antidiabetic agents, lipid‐lowering medications), baseline MMSE, and length of follow‐up.

Difference in cognitive trajectories over time (NPI‐Q and relative sub‐items change) between patients using AChEI and those not using AChEI were estimated using mixed‐effects repeated measures models of unstructured‐variance– covariance matrix. To formally test the hypothesis of different NPI‐Q and relative sub‐items slops over time the interaction term time*AChEI treatment was fitted in the model. The model was adjusted for age, sex, education, number of medical follow‐up visits, and follow‐up duration.

Comparisons between groups and time points were performed for the NPI‐Q total score and its subdomains. Given the exploratory nature of subdomain analyses and the intercorrelation among NPI‐Q items, no formal correction for multiple comparisons was applied. Findings for single subdomains should therefore be considered descriptive.

Statistical significance was defined as *p* < 0.05. Statistical analyses were performed using SPSS package version 21.0 (SPSS, Chicago, IL, USA).

## 3 Results

### 3.1 Population

**FIGURE 1:**
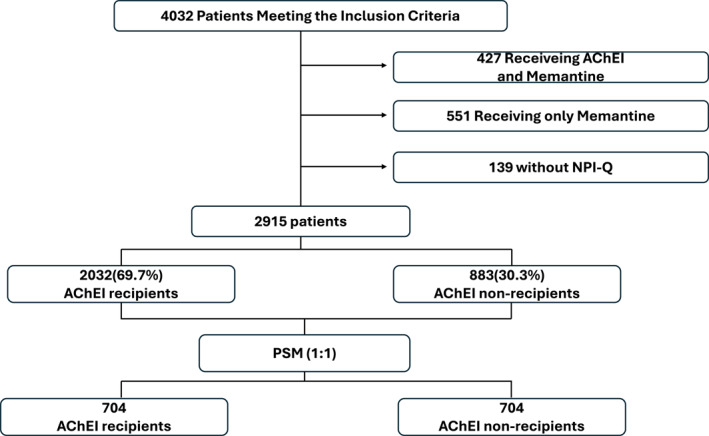
Flow chart of the study.

Overall, 4032 patients met the inclusion criteria. However, 427 patients who were treated with AChEI and memantine, 551 patients who were treated with memantine alone, and 139 patients who were missing the NPI‐Q assessment were excluded (Figure 1). The population included, at this point, 2915 patients who were initially divided into two groups, based on the presence or absence of AChEI therapy; 69.7% (2032 patients) were treated with AChEI (AChEI+), while 30.3% (883 patients) were not treated (AchEI‐). However, the two groups showed significant differences in many baseline characteristics. Through PSM each AChEI + patient was matched to an AChEI‐ patient with a similar propensity score (i.e., the estimated probability that an individual will receive treatment based on baseline covariates). A cohort of 1408 patients (704 AChEI+ and 704 AChEI−) was then generated (Figure 1). The main characteristics of the population enrolled into the study before and after PSM are reported in Table 1 A and B.

**TABLE 1:** Principal characteristics of the population enrolled into the study before (Table 1A) and after (Table 1B) propensity score matching.

	1A—before PSM			1B—after PSM		
	AChEI+ *N* = 2032	AChEI− *N* = 883	*p*	AChEI+ *N* = 704	AChEI– *N* = 704	*p*
Age	71.4 ± 4.8	75.3 ± 2.8	**0.001**	73.3 ± 3.8	73.2 ± 4.4	0.66
Males, *n* (%)	998 (49.1)	526 (59.5)	**0.001**	354 (50.2)	357 (50.7)	0.85
Years of formal education	14.8 ± 3.4	14.2 ± 6.6	**0.001**	14.3 ± 4.2	14.3 ± 40	0.99
MMSE	26.3 ± 1.4	25.5 ± 1.9	**0.001**	25.8 ± 1.6	25.9 ± 1.4	0.21
Caregivers' age (years)	54.3 ± 8.7	57.2 ± 7.7	**0.001**	56.2 ± 6.3	56.4 ± 7.3	0.59
Caregivers' sex (females, %)	1325 (65.2)	756 (85.5)	**0.001**	368 (52.2)	382 (54.2)	0.45
Type of dementia						
LOAD, *n* (%)	1021 (50.2)	521 (59.0)	0.72	383 (54.4)	389 (55.2)	0.76
LBD, *n* (%)	477 (23.4)	161 (18.2)	**0.02**	103 (14.6)	98 (13.9)	0.70
VaD, *n* (%)	534 (26.2)	201 (22.7)	**0.04**	218 (30.9)	217 (30.8)	0.96
Comorbidity						
Atrial fibrillation, *n* (%)	173 (8.5)	87 (9.8)	0.25	51 (7.2)	54 (7.6)	0.77
Stroke, *n* (%)	215 (10.5)	95 (10.7)	0.87	68 (9.6)	63 (8.9)	0.65
Hypertension, *n* (%)	1119 (55.0)	493 (55.8)	0.69	363 (51.5)	369 (52.4)	0.73
Diabetes, *n* (%)	421 (20.7)	261 (29.5)	**0.001**	194 (27.5)	200 (28.4)	0.70
Hypercholesterolemia, *n* (%)	919 (45.2)	437 (49.4)	**0.03**	288 (40.9)	293 (41.6)	0.79
Therapies						
Anti hypertensives, *n* (%)	1003 (49.3)	504 (57.0)	**0.001**	173 (24.5)	182 (25.8)	0.59
Anti diabetics, *n* (%)	394 (19.3)	247 (27.9)	**0.001**	164 (23.3)	175 (24.8)	0.48
Lipid‐lowering, *n* (%)	891 (43.8)	426 (48.2)	**0.02**	303 (43.0)	310 (44.0)	0.70
Antidepressants, *n* (%)	506 (24.9)	274 (31.0)	**0.001**	180 (25.5)	191 (27.1)	0.49
Antipsycotics, *n* (%)	134 (6.5)	106 (12.0)	**0.001**	58 (8.2)	68 (9.6)	0.35
Anxiolytics, *n* (%)	237 (11.6)	96 (10.8)	0.53	78 (11.0)	74 (10.5)	0.76

*Note:* The bold values denote significant *p*‐values (*p* < 0.05).

The follow‐up of the patients had a mean duration of 4.3 ± 1.6 years, with a range between 2.2 and 8.3 years. At baseline (T0), the cohort consisted of 1408 patients, of whom 704 were treated with AChEI. At the first observation point (T1: 2.0 ± 0.4 years) AChEI + patients were 692, AChEI‐ 612; at the second (T2: 4.0 ± 0.3 years), AChEI+ patients were 674, AChEI‐ 577; at the third (T3: 6.2 ± 0.3 years) AChEI+ were 628, AChEI‐ were 522, while at the fourth and last (T4: 8.1 ± 0.2 years) AChEI+ were 605, AChEI‐ 496.

### 3.2 NPI‐Q During the Follow‐Up

**FIGURE 2:**
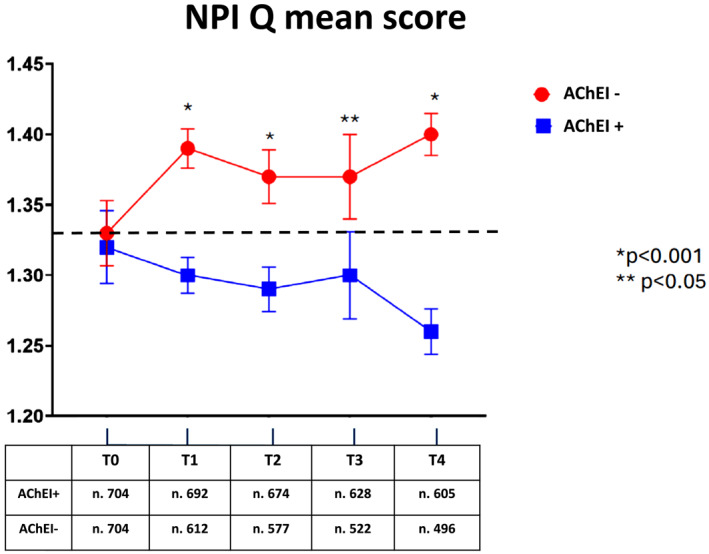
NPI‐Q changes during the follow‐up in patients treated (AChEI+) or not treated (AChEI−) with acetylcholinesterase inhibitors.

Over the follow‐up period (ranging from baseline [T0] to the fourth follow‐up [T4], with a maximum duration of 8.3 years), changes in the NPI‐Q total score and individual sub‐items were recorded and analyzed. As illustrated in Figure 2, the mean NPI‐Q score at baseline (T0) was 1.33 across the cohort. Over time, divergent trends were observed between the treatment groups: patients not receiving acetylcholinesterase inhibitors (AChEI−) showed a gradual increase in NPI‐Q scores (+6% from baseline), while those treated with AChEI (AChEI+) demonstrated a decrease (−6%). By T4, the mean score had risen to 1.41 in the AChEI− group and declined to 1.26 in the AChEI + group. These differences between the two groups reached statistical significance from T1 through T4 (Table 2).

**TABLE 2:** NPI Q and relative sub‐items scores at baseline (T0) and at the end (T4) of the follow‐up (only the sub‐items affected by AChEI treatment are reported).

	T0 SCORE		T4 SCORE	Absolute score change T0–T4 (range 0–3)	Group change T0–T4	Total change T0–T4
NPI Q	1.33	AChEI−	1.41	0.15	+6%	12%
		AChEI+	1.26		−6%	
HALL	1.75	AChEI−	20	0.25	+14%	14%
		AChEI+	1.75		0%	
AGIT	1.13	AChEI−	1.16	0.16	+3%	15%
		AChEI+	1.0		−12%	
AGGR						
DEPR	0.82	AChEI−	0.85	0.16	+4%	20%
DYSP		AChEI+	0.69		−16%	
ANX	10	AChEI−	1.1	0.22	+1%	13%
		AChEI+	0.88		−12%	
DISIN	1.05	AChEI−	1.5	0.70	+50%	74%
		AChEI+	0.8		−24%	
IRR	0.8	AChEI−	0.95	0.35	+18%	43%
LAB		AChEI+	0.6		−25%	

**FIGURE 3:**
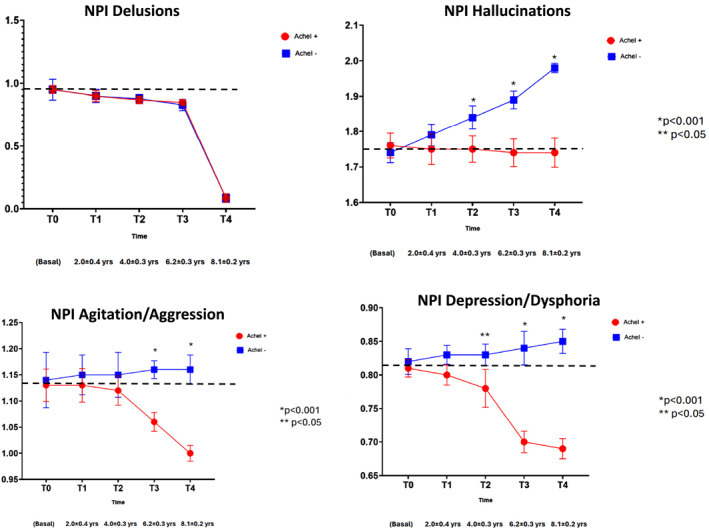
NPI‐Q sub‐items (delusion, hallucinations, agitation/aggression and depression/dysphoria) changes during the follow‐up in patients treated (AChEI+) or not treated (AChEI−) with acetylcholinesterase inhibitors.

**FIGURE 4:**
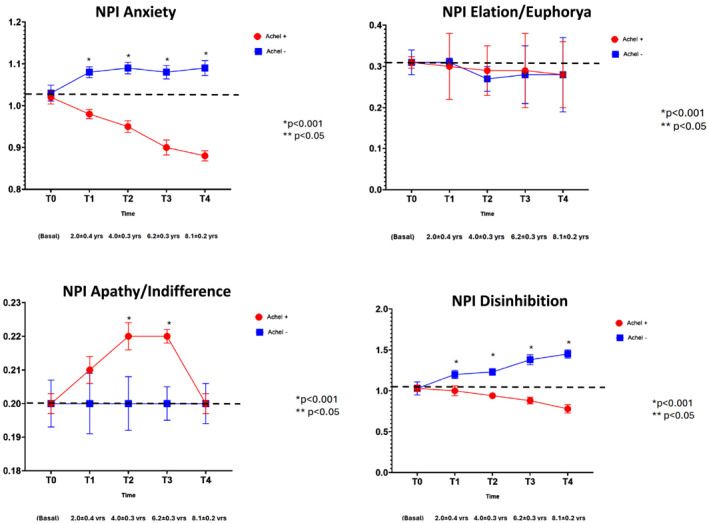
NPI‐Q sub‐items (anxiety, elation/euphorya, aphaty/indifference, and disinhibition) changes during the follow‐up in patients treated (AChEI+) or not treated (AChEI−) with acetylcholinesterase inhibitors.

**FIGURE 5:**
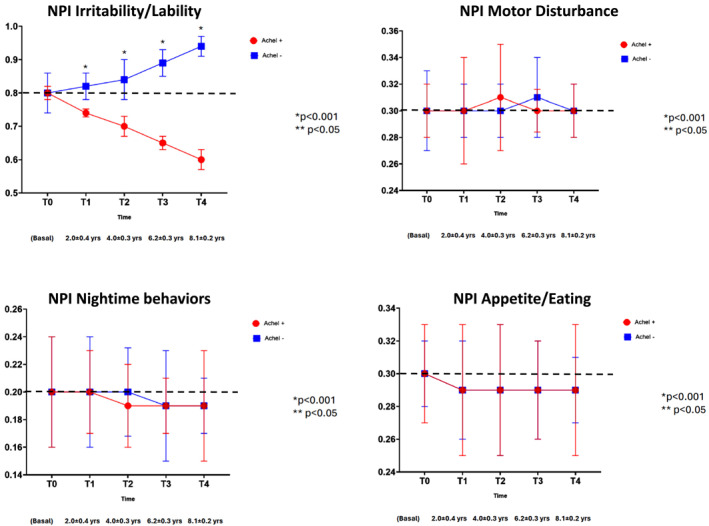
NPI‐Q sub‐items (irritability/lability, motor disturbance, nightime behaviors, and appetite/eating) changes during the follow‐up in patients treated (AChEI+) or not treated (AChEI‐) with acetylcholinesterase inhibitors.

When examining the NPI‐Q sub‐items, no statistically significant differences between AChEI− and AChEI+ groups were found for five domains: delusions, elation/euphoria, motor disturbances, night‐time behaviors, and appetite/eating changes (Figures 3, 4, 5). For apathy, a transient increase in scores was observed in the AChEI+ group at T2 and T3 compared to AChEI−, although this difference was no longer present at T4.

In contrast, six NPI‐Q sub‐items exhibited significant group differences over time, including hallucinations, agitation/aggression, depression/dysphoria, anxiety, disinhibition and irritability/lability.

### 3.3 Hallucinations

At T0, the mean score was 1.75. The average score of this subdomain was higher in LBD (2.05) and VaD (1.61) compared with AD (1.28). Similarly, the prevalence of hallucinations (NPI score > 0) was higher in LDB (48.8%) and VaD (31.9%) compared with AD (21%).

Over time, the AChEI− group experienced a 14% increase, reaching 2.0 at T4, while scores remained stable in the AChEI + group. The between‐group difference at T4 was 14%, with statistically significant differences observed from T2 to T4.

### 3.4 Agitation/Aggression

Starting from a baseline of 1.13, scores in the AChEI− group increased slightly (+3%), reaching 1.16 at T4. In contrast, the AChEI + group experienced a 12% reduction, reaching a mean score of 1.0. Statistically significant differences between the groups emerged at T3 and T4, with a 15% difference at the final time point.

### 3.5 Depression/Dysphoria

Baseline scores averaged 0.82. By T4, the AChEI− group showed a modest increase (+4%) to 0.85, while the AChEI + group demonstrated a notable decline (−16%) to 0.69. Group differences were significant at T3 and T4, with a 20% disparity observed at T4.

### 3.6 Anxiety

With an initial score of 1.0, the AChEI− group showed a minimal increase (+1%) to 1.1 at T4. Meanwhile, the AChEI + group saw a decrease of 12%, ending at 0.88. These differences were statistically significant from T1 through T4, with a 13% difference at T4.

### 3.7 Disinhibition

At baseline, both groups had a mean score of 1.05. Over time, the AChEI− group experienced a sharp increase (+50%) to 1.50, while the AChEI + group declined by 24%, reaching 0.80. This resulted in a substantial 74% difference between the two groups at T4, with significant differences observed from T1 onward.

### 3.8 Irritability/Lability

The baseline score of 0.80 rose by 18% in the AChEI− group (to 0.95), while the AChEI + group showed a 25% reduction, falling to 0.60 by T4. Statistically significant differences between the groups were present from T1 to T4, with a 43% gap at the final time point.

In Table S1 are reported the single NPI‐Q subitem unadjusted slopes (Δ T0 ‐ T4) according to dementia type (LOAD, LBD or VD); the analysis was only an “exploratory” due to insufficient sample size at later follow‐up waves (T3–T4). However, the qualitative pattern in different types of dementia consistently mirrored the primary findings; while AChEI + showed overall stabilization/modest improvement, AChEI‐ tended to exhibit progressive worsening over time.

## 4 Discussion

The analysis of data from the NACC UDS enabled us to investigate the potential long‐term effects of AChEIs on BPSD over an average follow‐up period of 4.3 years. Our study included a large cohort of individuals diagnosed with mild to moderate dementia, encompassing LOAD, VaD, and LBD.

One of the initial observations emerging from our data is that, overall, the severity of BPSD at baseline (T0) was relatively low. The mean total NPI‐Q score at T0 was 1.33 out of a maximum of 3, suggesting that, for most patients, behavioral symptoms were mild. A notable exception was the hallucinations sub‐domain, which had a baseline score of 1.75 (intermediate severity), higher than what usually reported in AD populations with comparable MMSE scores. Several factors may contribute to this finding: 1. we included patients with LBD and VaD (after PSM more that 40% of the sample had LBD or VaD) which present higher rates of visual hallucinations/psychotic symptoms compared to AD; 2. diagnostic overlap between AD and LBD is common in real‐world clinical settings, and neuropathological studies have shown that mixed AD/LBD pathology frequently manifests with hallucinations at relatively preserved cognitive levels; 3. The NACC UDS relies on multiple centers which may introduce some heterogeneity in diagnostic labeling, particularly in cases where subtle extrapyramidal/REM sleep‐behavior disorder features were present but not formally coded as LBD; 4. NPI‐Q hallucination subscale may capture a spectrum of perceptual disturbances (including visual misperceptions) which may inflate the average score relative to more restrictive definitions of hallucinations.

When comparing the AChEI + group with the AChEI− group, a general trend emerged; patients receiving AChEI therapy tended to show improvement or stabilization of BPSD over time, whereas those not receiving treatment more frequently exhibited a worsening of symptoms. However, the magnitude and consistency of this effect varied significantly across different symptom domains. Some behavioral symptoms appeared to respond more favorably to AChEI therapy, while others showed little to no apparent benefit.

### 4.1 Domains Influenced by AChEI Treatment

Compared to patients not treated, those receiving AChEI therapy exhibited a distinct pattern in the evolution of specific BPSD domains. Notably, treatment was associated with a stabilization in the severity of hallucinations and disinhibition, and with clear improvements in agitation, depression, anxiety, and irritability. In other words, in domains where the AChEI− group showed significant clinical deterioration, the AChEI + group remained stable; conversely, in domains where the AChEI− group remained relatively stable, the AChEI + group demonstrated a significant symptom improvement. In absolute terms, the behavioral domains that appeared to benefit most from AChEI therapy were disinhibition, irritability, and hallucinations. When considering relative change from baseline, the most pronounced benefits were observed in disinhibition, irritability, and depression.

Regarding the timing of these therapeutic effects, differences between the AChEI+ and AChEI− groups became statistically significant as early as T1 (2‐year follow‐up) for the total NPI‐Q score, as well as for anxiety, disinhibition, and irritability. Benefits in hallucinations and depression emerged by T2 (4 years), while improvements in agitation became evident by T3 (6 years).

As previously suggested by Wynn et al. [15], these results could be explained by the central role of the cholinergic system in the regulation of cognitive and emotional processes. Indeed, AChEI increases the availability of acetylcholine in the synaptic space, improving transmission in brain areas involved in behavioral regulation, including the prefrontal cortex and the limbic system. The prefrontal cortex is crucial for inhibitory control and emotional management: increased cholinergic activity may therefore contribute to the reduction of agitation, disinhibition and irritability. Furthermore, the cholinergic system interacts with serotoninergic and dopaminergic circuits involved in the regulation of mood and anxiety, and this might explain the positive effect of AChEI on depression and anxiety.

### 4.2 Domains Not Affected by AChEI Treatment

In some symptomatic dimensions of BPSD, no statistically significant differences were observed between the two groups during the entire follow‐up, suggesting a lack of efficacy of AChEI. Symptoms related to delusions and euphoria showed no improvement in treated patients compared to the AChEI‐ group. This could be because these symptoms are more closely linked to dysfunctions in neurotransmitter systems other than cholinergic, such as dopaminergic and serotonergic, on which AChEI have a limited impact. Similarly, aberrant motor behaviors, sleep disturbances, and appetite/eating disturbances do not appear to respond significantly to AChEI. In these cases, it cannot be forgotten that, as highlighted by Bittner et al. [16], the treatment with AChEI in AD and Parkinson's dementia may be associated with side effects including insomnia, agitation, and appetite disturbances, further complicating the interpretation of the results. An additional factor to consider is the potential floor effect in BPSD domains with low baseline prevalence. For behavioral symptoms with baseline NPI‐Q scores close to zero (≤ 0.3), the ability to detect further improvement is limited, which may partially explain the apparent lack of AChEI efficacy in these domains. Similarly, this floor effect could contribute to the transient increase in agitation observed in the AChEI + group during early follow‐up, as minor fluctuations may appear more pronounced when baseline severity is minimal. Accounting for such floor effects is important when interpreting the differential impact of AChEI across various BPSD domains.

Although several studies have highlighted the role of AChEI in slowing cognitive decline and improving prognosis in patients with dementia [17, 18, 19], the efficacy of these drugs on BPSD remains a matter of debate. Some meta‐analyses previously published and focused on AChEI treatment in patients with dementia have considered the BPSD as a possible primary or secondary outcome.

Campbell et al. (9 studies; average length: 6 months) found that among patients with mild to severe AD, AChEI had a beneficial effect on reducing BPSD compared to placebo (weighted mean difference: −1.38 NPI‐Q points), but they concluded that the clinical relevance of this effect remained unclear [10]. Rodda et al. (14 studies; average length: 6 months) found statistically significant, albeit modest, differences in the change of NPI‐Q score between patients treated with AChEI and placebo. They underlined that the interpretation of the results was limited by methodological considerations such as low NPI‐Q scores at baseline, and BPSD being investigated only as secondary outcome [20]. Blanco‐Silvente et al. (43 studies; mean length: 3–9 months) investigated the effect of AChEI on all‐cause discontinuation, efficacy, safety and risk‐benefit for AD [21]. Meta‐analysis and meta‐regression showed no evidence of AChEI efficacy on BPSD in patients with mild‐moderate AD. Leund et al. (4 studies; mean length: 3–26 months) reviewed the possible effect of AChEI on both cognition and BPSD in Chinese patients affected by AD, VaD, or mixed dementia [22]. These authors concluded that the evidence on BPSD was conflicting since one cohort study suggested that AChEIs are linked to an increase in NPI score, while another suggested a trend toward improved BPSD. Jin and Liu conducted a systemic review and bayesian network meta‐analysis (146 randomized clinical trials) to assess the comparative efficacy and safety of both pharmacological and non‐pharmacological therapies for BPSD [23]. As regards NPI evaluation, donepezil and galantamine were superior to placebo (mean difference: −1.45 and −1.80, respectively). The authors concluded that the pharmacological intervention is the first choice for BPSD treatment, and that galantamine and donepezil may provide the most proper efficacy, especially considering their overall therapeutic effects on dementia [23]. Dou et al. made a comparative analysis (effectiveness and safety) of AChEI and memantine for AD (41 randomized trials) [24]. Sixteen studies also measured the mean changes on NPI, but the network meta‐analysis indicated that all interventions did not have significant effects on BPSD compared with placebo.

As highlighted by the extensive literature discussed above, the efficacy of AChEI in managing BPSD remains inconclusive. It is important to note, however, that the meta‐analyses have relied almost exclusively on randomized controlled trials comparing AChEI to placebo, with follow‐up durations typically limited to less than 2 years. In this context, such studies are not directly comparable to our analysis, which draws on real‐world data from the NACC database and benefits from a substantially longer follow‐up period (mean: 4.3 years; maximum: 8.3 years). This extended observational window allows for a more robust assessment of the long‐term evolution of BPSD severity during dementia.

Several other studies have investigated psychotropic medication as an indirect measure ‐ or “proxy” ‐ for the presence and management of BPSD. In line with this approach, our own data revealed that, prior to PSM, patients in the AChEI− group were more frequently prescribed antipsychotics and anxiolytics compared to those in the AChEI + group, further suggesting a higher burden of BPSD in untreated individuals.

Narayanan et al. performed a retrospective analysis on newly admitted residents of 452 nursing facilities [25]; they included 845 patients taking rivastigmine and 517 control patients. The initiation of antipsychotics was lower in rivastigmine group (8.6%) compared with the controls (17%); the risk of taking antipsychotics was almost double (relative risk: 1.86) in controls compared to the to rivastigmine group.

In a cross‐sectional study, Fereshtehnejad et al. evaluated data from the Swedish Dementia Quality Registry (SveDem) on 5.907 newly diagnosed AD patients who were registered in memory clinics [26]. They found that co‐medication with antipsychotics (O.R.: 0.55; 95%CI: 0.38–0.79) and anxiolytics (O.R.: 0.62; 95%CI: 0.46–0.84) was significantly lower in the AChEI + group compared with patients without anti‐dementia treatment.

Orsel et al. analyzed data from the MEDALZ cohort (Finland, 2005–2011) including all community‐dwelling persons receiving a clinically verified AD diagnosis (70.719 individuals) [27]. The use of psychotropic drug (especially antipsychotics and antidepressants) increased during AD; 4 years after AD diagnosis, 18.3% of patients received ≥ 2 psychotropic drugs. The use of AChEI was inversely associated with psychotropic polypharmacy.

Tan et al. investigated whether AChEI therapy prevents/delays the initiation of psychotropic medications in patients with AD or LBD from a cohort 17.763 individuals registered in the Swedish Dementia Registry from 2007 to 2015 [28]. Compared with matched controls, AChEI + displayed a lower risk of antipsychotic (HR: 0.85; 95%CI: 0.75−0.95) and anxiolytic (HR: 0.76, 95%CI: 0.72−0.80) initiation, but not antidepressants or hypnotics.

Finally, we cannot ignore the fact that almost 20% of AD patients were not receiving AChEI at baseline although they are recommended by guidelines for decades. As we have already had the opportunity to comment [18], the efficacy of AChEI has been questioned probably as a consequence of different factors: 1. It is uncommon to notice improvements in cognitive performance after starting AChEI; 2. The effects of AChEI mainly consists of a slowdown/stabilization of cognitive decline, and this has not emphasized enough; 3. An important heterogeneity in the rate of dementia progression exists among patients, and this compromises the possibility of understanding the real effect of AChEI on the single subject.

### 4.3 Limitations of the Study

Our study has several limitations. 1. Participants comprising the NACC UDS database represent a “convenience sample”, including clinical‐referrals and community‐based U.S.; therefore, they are not representative of the U.S. population, and this partially reduces the generalizability of the results; 2. we excluded patients with short follow‐up (< 2 visits; most had incomplete data); of consequence we don't have short‐term information about the possible effect of AChEI on BPSD; 3. Even though we have “equalized” the 2 groups through PSM, the effects of some residual confounding may have results in not firm conclusions; 4. we have reported the overall trends in BPSD severity over time, but we have no data on the individual response to AChEI, nor data on the single AChEI molecule prescribed, and this allow us to speculate only on a possible class effect and not on the single molecules; 5. The comorbidities we observed may be different from those affecting patients with dementia in other regions of the world, partially limiting the generalizability of our findings; 6. The analyses of NPI‐Q subdomains were only exploratory and not adjusted for multiple comparisons, and the possibility of type I error inflation cannot be excluded; however, the consistency in direction and magnitude of the observed effects across related domains supports the overall reliability of our findings.

## 5 Conclusion

Our study supports the potential role for AChEI in the management of BPSD, demonstrating a trend toward symptoms stabilization or improvement in patients with mild to moderate dementia, compared to untreated individuals. The observed effects were not uniform across all symptom domains; rather, AChEI appeared to exert beneficial effects particularly on agitation, depression, anxiety, irritability, and hallucinations, while other behavioral symptoms remained largely unaffected. The Literature on this topic presents conflicting findings, likely due to methodological variability among studies, differences in follow‐up duration, and heterogeneity in the severity and type of dementia among participants. From a clinical standpoint, given that AChEI are widely used to mitigate cognitive decline, their potential to alleviate BPSD could represent an additional therapeutic benefit, potentially enhancing overall dementia care. Despite the limitations, our study contributes valuable long‐term data to the field and reinforces the relevance of AChEI in the comprehensive treatment of dementia. These findings highlight the need for further research aimed at better characterizing the subpopulations and specific behavioral symptoms that may respond most favorably to AChEI therapy.

## Conflicts of Interest

The authors declare no conflicts of interest.

## Supporting information


**Table S1**: NPI‐Q subitem slopes (Δ T0 ‐ T4) according to dementia type.

## Data Availability

The authors have nothing to report.
