# Supplementary material for: Long‐Term Effect of Acetylcholinesterase Inhibitors on Behavioral and Psychological Symptoms of Dementia
Source: Int J Geriatr Psychiatry. 2026 Jan 22;41(1):e70195. doi: 10.1002/gps.70195 (PMC12826130; doi:10.1002/gps.70195)
Supplement: Supplementary file 1 — Table S1: NPI‐Q subitem slopes (Δ T0 ‐ T4) according to dementia type. [file GPS-41-e70195-s001.docx]

Supplementary TABLE 1: NPI-Q subitem slopes (Δ T0 - T4) according to dementia type.

|  | **Δ LOAD** | | **Δ LBD** | | **Δ VaD** | |
| --- | --- | --- | --- | --- | --- | --- |
|  | **AChEI -** | **AChEI +** | **AChEI -** | **AChEI+** | **AChEI -** | **AChEI+** |
| **Hallucinations** | +0.20 | 0.00 | +0.35 | –0.08 | +0.20 | 0.00 |
| **Agitation/Aggression** | +0.05 | –0.10 | +0.12 | –0.15 | +0.06 | –0.10 |
| **Depression/Dysphoria** | +0.04 | –0.12 | +0.08 | –0.16 | +0.05 | –0.10 |
| **Anxiety** | +0.02 | –0.10 | +0.06 | –0.14 | +0.03 | –0.08 |
| **Disinhibition** | +0.40 | –0.25 | +0.60 | –0.28 | +0.45 | –0.22 |
| **Irritability/Lability** | +0.15 | –0.20 | +0.30 | –0.25 | +0.18 | –0.18 |
